# Defaunation of large-bodied frugivores reduces carbon storage in a tropical forest of Southeast Asia

**DOI:** 10.1038/s41598-019-46399-y

**Published:** 2019-07-10

**Authors:** Wirong Chanthorn, Florian Hartig, Warren Y. Brockelman, Wacharapong Srisang, Anuttara Nathalang, Jantima Santon

**Affiliations:** 1 Department of Environmental Technology and Management, Faculty of Environment, Kasetsart University, 50 Ngamwongwan Road, Jatujak District, Bangkok, 10900 Thailand; 2 Theoretical Ecology, University of Regensburg, Universitätsstraße 31, 93053 Regensburg, Germany; 3 BIOTEC, National Science and Technology Development Agency, 113 Science Park, Paholyothin Road, Klong Luang, Pathum Thani 12120 Thailand; 4 Institute of Molecular Biosciences, Mahidol University, Salaya, Phutthamonthon, Nakhon Pathom, Thailand; 5 Rajamangala University of Technology Lanna, Faculty of Science and Agricultural Technology, Lampang, Thailand

**Keywords:** Biodiversity, Conservation biology, Tropical ecology

## Abstract

Recent studies have suggested that defaunation of large-bodied frugivores reduces above-ground carbon storage in tropical forests of South America and Africa, but not, or less so, in Southeast Asian tropical forests. Here we analyze the issue using the seed dispersal network (data of interaction between trees and animal seed dispersers) and forest composition of a 30-ha forest dynamics plot in central Thailand, where an intact fauna of primates, ungulates, bears and birds of all sizes still exists. We simulate the effect of two defaunation scenarios on forest biomass: 1) only primates extirpated (a realistic possibility in near future), and 2) extirpation of all large-bodied frugivores (LBF) including gibbons, macaques, hornbills and terrestrial mammals, the main targets of poachers in this region. For each scenario, we varied the population size reduction of the LBF dispersed tree species from 20% to 100%. We find that tree species dependent on seed dispersal by large-bodied frugivores (LBF) account for nearly one-third of the total carbon biomass on the plot, and that the community turnover following a complete defaunation would result in a carbon reduction of 2.4% to 3.0%, depending on the defaunation scenario and the model assumptions. The reduction was always greater than 1% when the defaunation intensity was at least 40%. These effect sizes are comparable to values reported for Neotropical forests, suggesting that the impact of defaunation on carbon deficit is not necessarily lower in Southeast Asian forests. The problem of defaunation in Asia, and the mutual benefits between biodiversity conservation and climate change mitigation, should therefore not be neglected by global policies to reduce carbon emissions.

## Introduction

Defaunation of large-bodied animals in the tropics is a global concern. Large animals are primary targets for hunting, particularly in areas with good transport connections to major towns where wild meat can be sold^1^. They are also highly vulnerable to forest fragmentation^2^. The resulting population declines are not only a concern because of the direct loss of the hunted species, but also because of indirect impacts on other trophic levels and ecosystem functioning associated with the decline of these animal groups^3^.

For plants, the most important consequence of defaunation is a loss of seed dispersal vectors. Trees that depend on large-bodied frugivores (LBF) for seed dispersal were more aggregated after only 15 years of defaunation in a large forest plot in Borneo^4^, and similar results have been reported from a Neotropical forest in Peru^5^. Despite the apparent high redundancy within the seed dispersal network of tropical forests, some large-bodied frugivores play crucial roles in maintaining species diversity^6^, and defaunation may thus have negative long-term effects on forest diversity. Furthermore, loss of dispersal effectiveness may occur long before the dispersal vectors are extinct, when populations of seed disperses become too small to be effective^7^.

The loss of species dependent on seed dispersal by LBF is not only a threat to forest biodiversity but could also scale up to ecosystem-level properties, such resilience against climatic variability, or carbon storage^3,8–12^. Ecological theory suggests that a loss of seed dispersal capacity can reduce ecosystem performance for at least three reasons: increased intraspecific competition, and decreased ability to find optimal environmental conditions, and trait differences between animal dispersed and other tree species. The first reason refers to the fact that reduced dispersal creates more clustered distributions of conspecifics, which may increase pressure from shared pathogens and predators^13,14^ that cause density dependent mortality^2,15,16^. The second point refers to the fact that a loss of mobility or dispersal limitation reduces the ability of organisms to colonize new areas^17^, and is worse when local habitat becomes less suitable, for example, through changes in soil or light availability during forest succession^18,19^ or as a result of logging^20^. The third point refers to the fact that animal-dispersed species may have different ecological strategies and properties, suggesting that their loss cannot be fully compensated by existing species in the ecosystem.

Assuming that the loss of important seed dispersers would also cause a loss of their associated tree species, several recent studies have predicted that defaunation, particularly of large-bodied frugivores, will reduce carbon storage in tropical forests^8–12^. The key element of these predictions is the finding of a positive association between LBF dispersal and high wood density (or wood specific gravity, WSG). Estimates of the reduction of carbon storage varies depending on region, from below 2% to 12% in Neotropical forests (e.g., Panama, Costa Rica, Brazil and other Amazonian countries), African (e.g., Cameroon, Congo and Tanzania) and Asian forests (e.g., India, Malaysia)^11,20^. These findings imply that forests cannot reach their maximum carbon storage potential without the wildlife species that make up their seed dispersal networks, which has important consequences for global carbon policies such as reducing carbon emissions from deforestation and degradation plus biodiversity conservation and sustainable development (REDD+) – the policy initiated by the United Nations Framework Convention on Climate Change (UNFCCC) that simultaneously promotes carbon sequestration and biodiversity conservation.

In Southeast Asia, defaunation and deforestation rates are as high as anywhere in the tropics^21^. Substantial declines in wildlife are occurring in the region due to illegal domestic and international trade of wildlife^21^. Moreover, deforestation greatly increased from 2000 to 2010, mostly due to conversion of forest area to cash crops, such as oil palm^22^. In contrast to the Neotropics, however, studies have so far found little evidence for a decrease in above-ground carbon due to defaunation. A possible explanation is the large share of wind-dispersed Dipterocarpaceae species in Asian forests, a family known for its high WSG. It has therefore been conjectured that biomass loss due to defaunation is of lesser importance in Asia than in the Neotropics^11^. Not all forests in Southeast Asia, however, are dominated by Dipterocarpaceae^23,24^, questioning the generality of the claim.

In this study, we investigate the potential impact of defaunation on carbon storage in a 30-ha forest dynamics plot in Thailand^23^. The study site, located in a national park, has not been subjected to recent hunting or logging. Consequently, the density of wildlife is relatively high, and detailed studies of the diets of some of its large frugivores, including primates, deer, bears, hornbills and smaller birds (see Brockelman *et*. *al*.^23^) have been conducted. Large-bodied frugivores that occur on the plot, such as Asian elephant, sambar deer and bears, have extensive home ranges. This makes them important seed dispersers on a landscape scale in Southeast Asian forests^25–28^, in particular for some important old-growth tree species with high WSG such as *Garcinia* spp.^29^ and *Platymitra macrocarpa*^30^. The intact seed dispersal network and the large size of the plot minimize limitations associated with other studies, in particular detailed knowledge about the dispersal network, and variability due to the low numbers of large trees normally found in studies on small plots^31^, making the plot ideal for studying the benefits of frugivores to trees in this region.

## Results

Analyzing the plot data, we find that tree species dependent on seed dispersal by large-bodied frugivores account for nearly one third of the total (30 ha) plot above ground carbon (AGC) of 4,559.2 tons (160 Mg/ha, Fig. 2). The AGC of species dispersed by primates alone accounted for about 13%, and species dispersed by terrestrial mammals combined or large-bodied birds for about 21%, of the total AGC. Median WSG was significantly higher in tree species dispersed by LBF than for those dispersed by other animals (*t* = 2.08, *P* < 0.05), while maximum tree size was not significantly different between those groups (*t* = 1.35, *P* > 0.1; Fig. 3; see also Supplementary Table S2). We found the same pattern when we concentrated on primates only (WSG: *t* = 2.01, *P* < 0.05; maximum size: *t* = −0.94, *P* > 0.3; Fig. 3; see also Supplementary Table S2). Among the animal-dispersed tree species, maximum tree size was slightly higher for non-primate dispersed species, possibly because the most important primate fruits are canopy and middle canopy species, but not tall emergent species; however, this difference was not significant.

**Figure 1:**
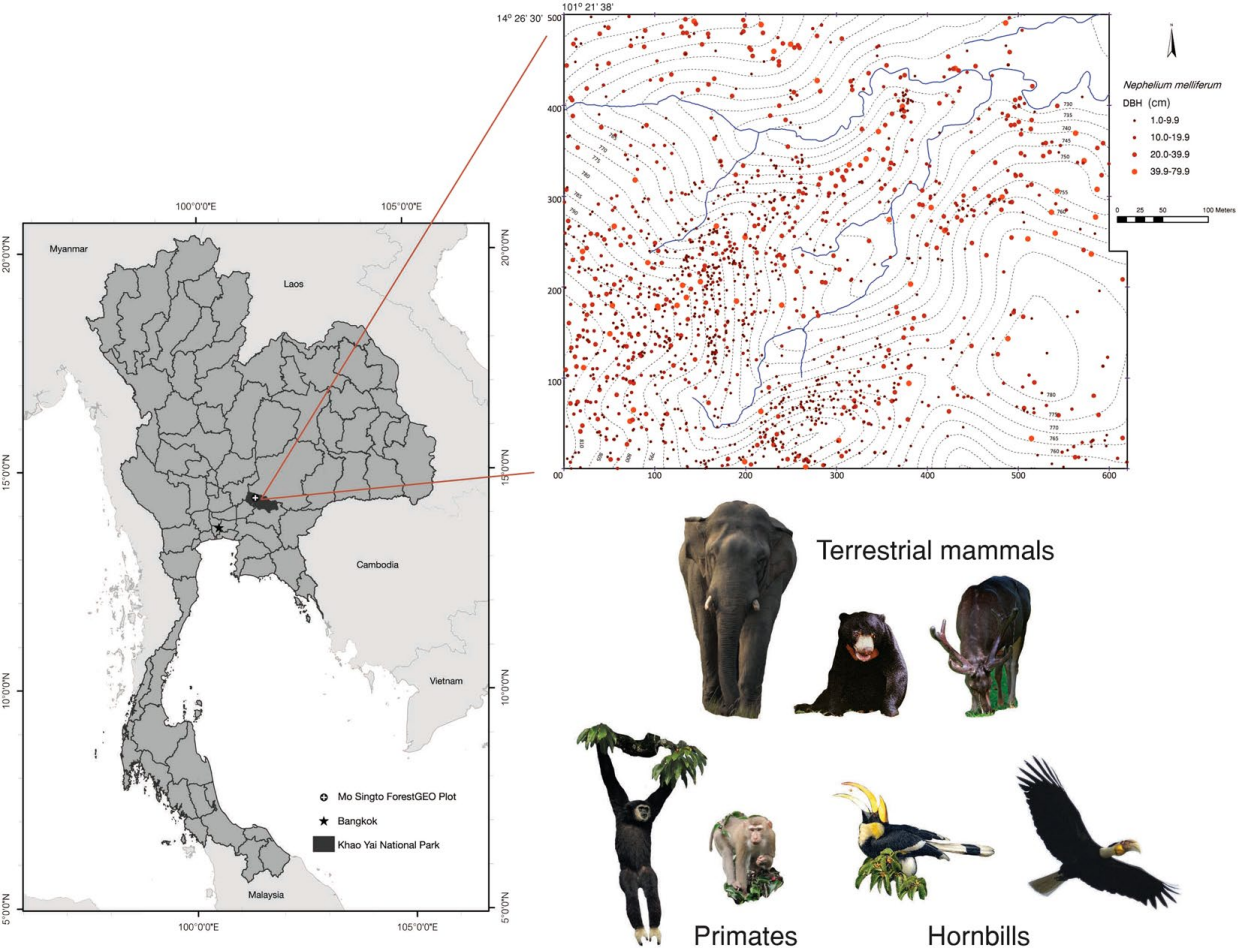
Geographic location and topographic map of the Mo Singto ForestGEO plot as well as the group of large-bodied frugivores frequently observed in the plot. The topographic map also shows the distribution of *Nephelium melliferum*, a hardwood canopy species dispersed mainly by primates.

**Figure 2:**
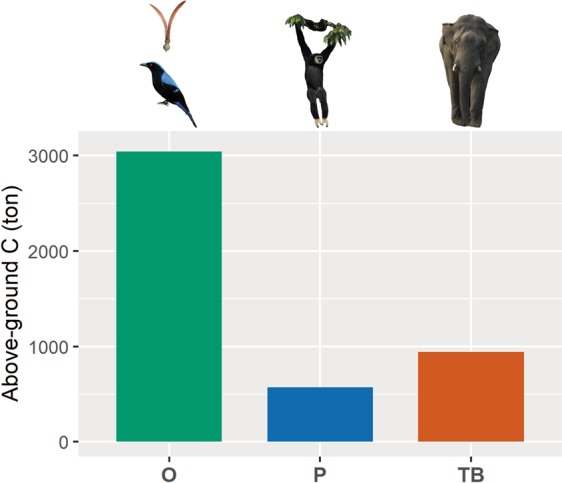
Share (%) of total above ground carbon (4,559.2 ton C/30 ha), considering only trees with dbh ≥5 cm categorized by dispersal modes (P is primates, TB is large-bodied terrestrial, mammals and birds, and O is other seed dispersal agents, mostly smaller birds and abiotic).

**Figure 3:**
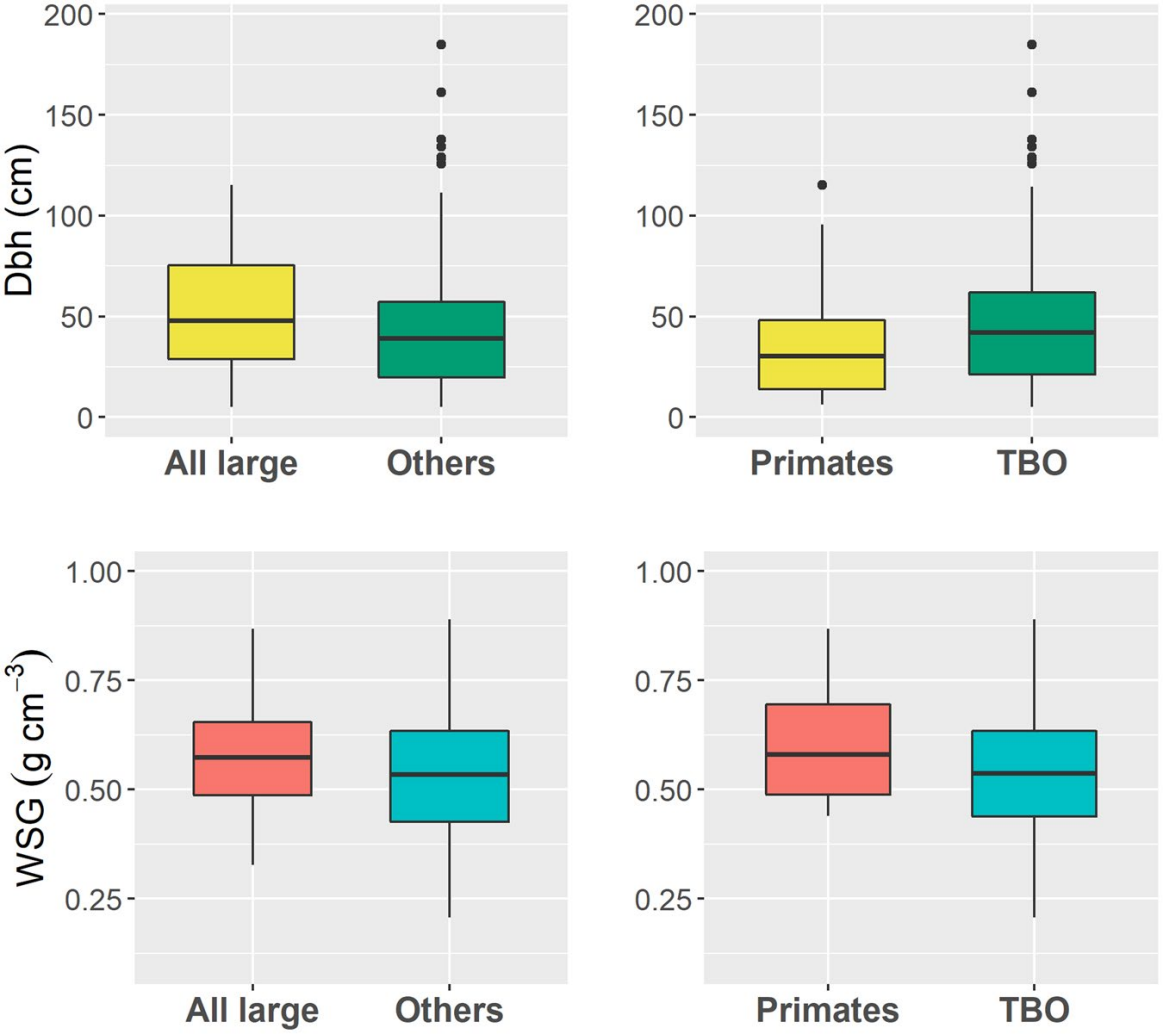
Maximum size and wood specific gravity: compared between trees species dispersed by all large-bodied frugivores (All large) versus the other agents (Others; left panels), and compared between primate-dispersed species (Primates) versus species dispersed by terrestrial mammals, birds and other large dispersers (TBO; right panels).

**Figure 4:**
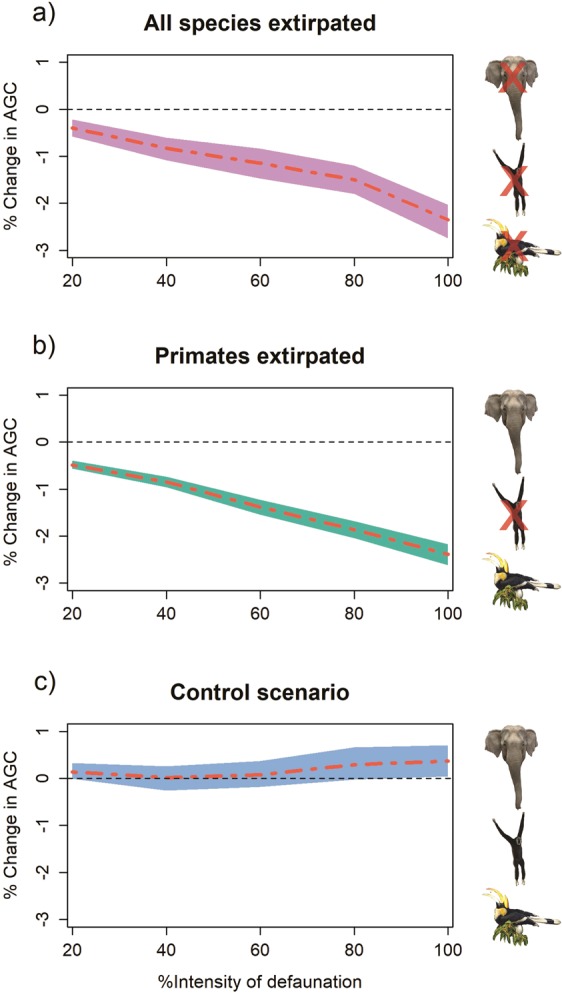
Percent change in above-ground carbon (AGC) storage with 95% confidence interval bands in three scenarios: (**a**) all individual trees dispersed by LBF are extirpated, (**b**) only primates are extirpated and (**c**) control scenario—all individual trees have equal chance of extirpation, across the gradient of defaunation intensity (see values in Supplementary Tables S3–S6).

Our simulation results show that carbon storage would decline under both (all frugivores, or only primates extirpated) defaunation scenarios (Fig. 4). In both scenarios, the AGC declined more than 1% at 40% defaunation, and approximately 2.4% at 100% defaunation. When we simulated based on the assumption of constant size structure AGC was reduced by 3.02% and 2.31% for the reduction of all LBF and only primates, respectively (see Supplementary Tables S3, S4). In the constant size structure model, the primate scenario had a lower effect relative to the LBF scenario because the maximum sizes of trees dispersed by primates are lower than for all LBF, as discussed above. The confidence intervals calculated from a randomization of the simulation were relatively narrow, so that a local reduction of AGC by at least 2% for both scenarios with 100% defaunation is statistically reliable, provided the assumptions of the model hold (Fig. 4, see Supplementary Tables S5, S6). We make further comments on factors contributing to uncertainty of our estimates in the see methods section.

## Discussion

Several recent studies have argued that defaunation in tropical forests of Southeast Asia will have lower effects on carbon storage than in other tropical biomes, because of the prevalence of hardwood, large-boled, wind-dispersed Dipterocarpaceae in this region^4,11^. Our results, however, suggest that this may not be true for all Asian forests. For our study site in east-central Thailand (Fig. 4), where only one dipterocarp species (*Dipterocarpus gracilis*) is common, our simulations suggest that defaunation could cause a biomass reduction of 2.4% to 3.0%. While this value is in the lower range of other defaunation predictions globally, it is similar in magnitude to results reported from recent studies in Neotropical forests, which found values below −2%^8,10,11^. We assume that a lower share of dipterocarp species may be a key factor for the greater loss in AGC through defaunation in our study. Generally, most dipterocarp species in mature forest are emergent trees. They tend to be taller than other species at the same diameter. They also tend to have higher mean wood specific gravity (WSG) than the community mean. If they successfully colonize, their AGC may compensate for the loss of high WSG species dispersed by LBF, or even increase the carbon storage in a forest (see the simulation results by Osuri *et*. *al*.^11^). In our plot, dipterocarp species comprise only 13% of the plot’s total basal area, while the share on other large plots in Southeast Asia is 28% (Pasoh), 41% (Lambir) and 21% (Huai Kha Khaeng)^32^. The center of diversity for Dipterocarpaceae is in the Malesian forests of Sundaland^33^ Lower diversity occurs in the Indo-China region, which our plot borders, and where LBF-dispersed species belonging to families such as Anacardiaceae, Annonaceae, Meliaceae, Myristicaceae, Myrtaceae and Sapindaceae^24^ are more commonly found found. It would be interesting to gain a better understanding of the distribution patterns of LBF-dispersed species and genera in Asia and around the world, and relate this to regional differences in defaunation impacts and predictions.

WSG and tree size (usually measured as diameter at 1.3 m height) are essential components for calculating forest biomass^34,35^, together with community size structure. Our simulations based on these traits predicted a statistically significant biomass reduction under an assumption of complete or partial defaunation of the current community. The dominant part of this reduction is most likely due to differences in wood density (there is a significant association between wood density and species dispersed by LBF vs. other modes as well as by only primates vs. other modes; Fig. 3). These results mirror the patterns found in Neotropical forests, where WSG is usually positively correlated to seed size^8,10,11^. However, further studies are necessary for a better understanding of the generality and possibly spatial patterns of this association. Studies are also needed to better understand the characteristics of species important for seed dispersal, such as fruit characteristics and seed dispersal effectiveness, as well as secondary seed dispersal^17^.

Our estimate of carbon reduction from defaunation was derived essentially by recreating the forest community without animal dispersed species, assuming that the share and size structure of the remaining species remain stable. However, as species compete with each other in complex ways, there is considerable uncertainty associated with this assumption. Contributors to more complex reactions could be the varying degrees of “seed dispersal effectiveness”^36^ across scales among LBF such as gibbons, macaques and hornbills that disperse *Prunus javanica* in the Mo Singto plot^37^, or different density-dependence in LBF and non-LBF species. A recent study by Bagchi *et al*.^5^, for example, found no density-dependent mortality in species dispersed by LBF in a Peruvian forest. Such differences in density dependence between animal-dispersed and other species may positively or negatively affect the impacts of defaunation.

Despite these uncertainties, we believe that our results provide a fairly robust estimate of a potential biomass loss through defaunation. Evidence for this is provided, firstly, by the control runs that applied the same resampling steps without removing the animal-dispersed species, which resulted in a slight increase in biomass, suggesting that our simulation steps do not introduce a bias in favor of a reduction of biomass. Secondly, we assumed in our simulation that the remaining tree species can essentially take over the roles of the animal-dispersed species in the forest community. This is a rather optimistic scenario, assuming that functional redundancy ensures that all niches and roles filled by animal-dispersed species can be taken over by non-animal-dispersed species. If that is not the case, biomass reductions could be much stronger. Thirdly, a loss of tree diversity may increase intra-specific competition or other causes of density dependent mortality^2,15,16^, such as increasing of pressure from shared pathogens and predators^13,14^, and consequently lead to further reduction of AGC. A limitation of our model (as well as those of other studies^8,18,20^) is that it assumes a community equilibrium. Modelling the transient dynamics and extinction debt for this plot (i.e. population dynamics of a species-rich tropical forest with >260 species) would, however, require a large dynamic model, a dauntingly complex task even without incorporating the loss of seed dispersers. Without dedicated empirical data for each species (cf. Caughlin *et al*.^15^), it seems impossible to build such a model. Another limitation of our model is the possibility of secondary seed dispersal by terrestrial animals such as rodents, which may be able to compensate for defaunation of LBF to a certain degree^33,36^. The possibility of this compensation is, however, is partly addressed by our varying degree of population size reduction in the simulations.

Our results imply that REDD+ ^38^ and other conservation programs in Southeast Asian tropical forests should pay more attention to defaunation. Large-bodied animal populations are considerably reduced in many areas, and often critically endangered^3,16^. Southeast Asia has a particularly severe problem of defaunation, and many species have already become regionally extinct^21^, or else functionally extinct in their communities^7^. Global policies, particularly REDD+ and forest restoration projects, should therefore better account for the fauna and interactions with plants such as seed dispersal in order to maintain biodiversity^2,6^ and ecosystem services^3^ in tropical forests.

## Methods

### Study site

We used data from the 30-ha Mo Singto forest dynamics plot (Fig. 1), a ForestGEO plot in the network of the Center for Tropical Forest Science, Smithsonian Tropical Research Institute (forest-geo.si.edu). The plot is located at 101°22′ E and 14°26′ N in Khao Yai National Park, Thailand, at 725–815 m altitude (see the map in Fig. 1). The average annual precipitation is *ca*. 2,100 mm and average annual minimum-maximum temperatures range over 19°–28 °C. All trees on the plot with dbh ≥1 cm are tagged and mapped. In this study we used data from the third census conducted in 2010. The forest type has been classified as northern seasonal evergreen forest, whose main distribution is in southern China (e.g., Xishuangbanna) and northern Laos and Vietnam^33^. The flora of the plot is reported in Brockelman *et al*.^23^. Since Khao Yai National Park was established in 1962, there has been relatively little anthropogenic disturbance such as hunting and logging (except for poaching of a few high-value species such as *Aquilaria crassna*, which produces aromatic gharuwood).

### Seed dispersal and large-bodied frugivore classification

Since hunting pressure has been very low for at least 55 years in the region around the park headquarters, several large-bodied frugivores (LBF), most on the IUCN red list (http://www.iucnredlist.org), are still common in the plot (Fig. 1), for example the Endangered white-handed gibbon (*Hylobates lar*) and Asian Elephant (*Elephas maximus*), and the Vulnerable pig-tailed macaque (*Macaca leonina*), Asiatic black bear (*Ursus thibitanus*), Malayan sun bear (*Helarctos malayana*), sambar deer (*Rusa unicolor*), great hornbill (*Buceros bicornis*) and brown hornbill (*Anorrhinus austeni*). Other important large-bodied frugivores categorized as Least Concern by IUCN include wreathed hornbill *(Rhyticeros undulatus*), Oriental pied hornbill (*Anthracoceros albirostris*), mountain imperial pigeon (*Ducula badia*)^39^. Although the brown and pied hornbills and imperial pigeon are medium sized and not large frugivores, they are included because their gape allows them to consume many of the fruits consumed by large-bodied frugivores.Although they are not all globally Endangered species, they are locally susceptible to hunting and have been extirpated from all non-protected areas in Thailand.

Seed dispersal agents of each tree species were categorized based on literature from the area^27,40–48^, our herbarium (Biotec Bangkok Herbarium) and our unpublished databases, which include information on fruit and seed characteristics and consumers of nearly all fruiting trees in and around the Mo Singto plot. Frugivores were classified into eight groups (see Supplementary Table S1): (1) gibbon, (2) macaque, (3) hornbills and large pigeons, (4) smaller birds, (5) terrestrial mammals (deer, elephants and bears), (6) unknown animals, (7) unclassified or unknown dispersal mode (including ballistic), and (8) wind. We defined LBF as gibbon, macaque, hornbills and terrestrial mammals, the main targets of poachers in this region. The diets of gibbons and pig-tail macaque in the Mo Singto forest are known in considerable detail^41,47,48^, and we have identified a suite of “primate fruits” that typically have covers or rinds that prevent them from being consumed by birds. There is a fairly consistent difference in the average seed sizes of tree species dispersed by LBF and the other agents with a seed length cut-off at approximately 15 mm^40^. It was assumed that species consumed by LBF would succumb to defaunation due to dispersal limitation, and if not completely extirpated, would become rare and largely nonfunctional ecologically (see Supplementary Table S1).

Civets (particularly common palm civet, *Paradoxurus hermaphroditus*) are relatively large omnivorous species present in our study area. Their gape width is large enough to consume seeds as large as those of LBF^49^, and reports from Southeast Asia have shown their dietary overlap LBF^50^. As knowledge of seed dispersal by civet species is limited in our study area, we did not explicitly include them in this study. We assumed that they share their fruit diet with other LBF. This assumption may affect the scenario of primate extirpation as described in the later subsection, but not in the scenario of all LBF extirpation. However, we assume that their effect is negligible in our simulations, because they rarely feed on typical primate fruits such as *Nephelium melliferum* in our plot (unpublished data) and frequently disperse seeds out of old-growth forest into disturbed areas^49,51,52^.

### Wood specific gravity and maximum tree size comparison

Because wood specific gravity and maximum tree diameter at breast height (dbh) are the key traits affecting biomass estimation, we tested differences of mean between the species dispersed by LBF and other dispersal agents using t-tests. Wood density values were derived mainly from the global wood density database^53^. If a species present in the Mo Singto plot was not contained in the database, we used the median of all species in the genus or family. However, we also sampled some tree species that had no species-level data available in the global database from surrounding areas of the plot. We therefore used this data instead of using the mean WSG at genus level for these species.

### Calculation of above-ground carbon

As we aimed to compare with the study in Southeast Asia, we therefore estimated above-ground carbon (AGC) from above-ground biomass with the same equation as Osuri *et al*.^11,54^:

$$AGB=\rho \times \exp {(-1.499+2.148\mathrm{ln}(D)+0.207{(\mathrm{ln}(D))}^{2}-0.281(\mathrm{ln}(D))}^{3})$$

Where *ρ* is the wood density (g/cm^3^) and *D* is dbh (≥5 cm). For presentation of the final results as AGC, we approximated the above-ground carbon as half of above-ground biomass as also applied in Osuri *et al*.^11,54^.

### Simulation of defaunation results

For simulating defaunation, we used all trees with dbh ≥5 cm, which resulted in 33,844 individuals of 232 species in the 30-ha plot. To arrive at an estimate of defaunation impacts, we followed the same principles as previous analyses from the Neotropics^8,10,11^, using an abundance-weighted stochastic model. The general idea is that tree species dispersed by LBF are removed (extirpated due to seed dispersal limitation) and replaced (colonized) by other tree species from the community. The simulation makes zero-sum assumptions, meaning that the total community size, *N*_*T*_, is fixed. We further subdivided *N*_*T*_ into four diameter classes, 5–20, 20–40, 40–80 and >80 cm, for each of which simulation was made using zero-sum assumptions. The stratification will approximately ensure an identical size structure after replacement. Small differences could occur if there are systematic differences between the size structure of LBF and non- LBF species. The latter is conceivable, as strata distribution or vertical niche is an important life history trait for tropical tree species, which might possibly correlate with dispersal ability. To be able to control for this possibility, we considered two simulation options: The first option is to not completely replace LBF species, but to only change their wood specific gravity to the density of a random sample of non-LBF. This procedure is similar to the simulations conducted by Bello *et al*.^20^ and Osuri *et al*.^8^, in which we ensure that both size (basal area) and the number of individuals remain constant. The second option is replace all individuals, including their size, as in the procedure of Peres *et al*.^18^ We ran both options, because we think both are ecologically defensible. The first option assumes that only wood densities change, but size structure will remain constant after defaunation. The second option assumes that size structure is an inherent property of the LBF and non-LBF species, and will thus also change if one of the groups is removed. Based on our detailed data on the dispersal network, we allowed that not only wind-dispersed species, but also tree species dispersed by small-bodied frugivores (birds, bats, rodents) could replace LBF-dispersed species, similar to defaunation scenarios with compensation in Bello *et al*.^10^

From the simulation results, we calculated the change of AGB by subtracting the simulated data from the original observed census data. We ran the simulation 200 times. To generate confidence intervals at 95%, we bootstrapped these steps 200 times as well.

We examined three scenarios based on extinction-risk status in the IUCN’s Red List. The first scenario was the extirpation of only the primates (the Endangered gibbon *Hylobates lar* and the Vulnerable pig-tailed macaque *Macaca leonine*), which we consider a realistic possibility for the near future. *H*. *lar* disperses many tree species with high carbon content (high WSG) inside mature or old-growth forest^40,46^ (see Supplementary Table S1). *M*. *leonina*, on the other hand, is a generalist seed predator that forages across many habitat types, including grassland and human-used areas^40,41^. The two species nevertheless have shared links to many fruit species in our plot^23,40^. The second scenario was that all LBF are extirpated. This scenario is more extreme and not very likely in the national park, but may be realistic in landscapes with poorer protection and strong human pressure^10^.Thirdly, we tested a control scenario, in which individuals of all tree species were randomly removed and replaced in the same number as run in the first scenario (all LBF extirpated). This control scenario tests for the effect of an artefact due to only the procedure involved.

In each scenario, we explored a gradient of defaunation intensity, similar to Bello *et al*.^10^ and Osuri *et al*.^11^. We varied the effective intensity of defaunation in five steps: 20%, 40%, 60%, 80%, and 100%. The 100% intensity is the case that all individuals of tree species dispersed by LBF are removed; for the other cases only the respective fraction is removed. For example, 40% means removal of 40% of the number of trees dispersed by LBF.

## Supplementary information

Supplementary Information

## Data Availability

Species and their dispersal modes are included in this published article (and its Supplementary Information File). The census data is available at CTFS-ForestGEO Data Management. The R codes of all analyses are available at https://github.com/Wirong/Defaunation.
