# Supplementary material for: Defaunation of large-bodied frugivores reduces carbon storage in a tropical forest of Southeast Asia
Source: Sci Rep. 2019 Jul 10;9:10015. doi: 10.1038/s41598-019-46399-y (PMC6620352; doi:10.1038/s41598-019-46399-y)
Supplement: Supplementary file 1 — Supplementary Information [file 41598_2019_46399_MOESM1_ESM.pdf]

Supplementary information for

Defaunation of large-bodied animals reduces carbon storage in a tropical forest of Southeast Asia

Wirong Chanthorn[1,*], Florian Hartig[2], Warren Y. Brockelman[3,4], Wacharapong Srisang[5], Anuttara Nathalang[4], Jantima Santhon[4]

[1]Department of Environmental Technology and Management, Faculty of Environment, Kasetsart University, Ngamwongwan road, Bangkok 10900, Thailand. [2] Theoretical Ecology, University of Regensburg, Universitätsstraße 31, 93053 Regensburg, Germany. [3]Ecology Laboratory, BIOTEC, 113 Science Park, Paholyothin Road, Klong Luang, Pathum Thani 12120, Thailand. [4]Institute of Molecular Biosciences, Mahidol University, Salaya, Phutthamonthon, Nakhon Pathom, Thailand. [5]Rajamangala University of Technology Lanna, Faculty of Science and Agricultural Technology, Lampang, Thailand.

Correspondence and requests for materials should be addressed to W.C. (email: fsciwrc@ku.ac.th)

Table S1 All species employed in the analyses of this study with information of strata (Str), maximum dbh size (cm, Max dbh), wood specific gravity (g/cm^3,WSG) fruit types, ripe color, and dispersal agents (i.e., Gi=gibbon, Ma=Macaque, Ho=hornbills, SB=smaller birds, An=Unknown animals, TM=Terrestrial mammals, Wi=wind, Un=unknown).

Family	Species	Str	Max dbh	WSG	Fruit types	Ripe color	Gi	Ma	Ho	SB	An	TM	Wi	Un
Fabaceae-caesalpinioideae	*Acrocarpus fraxinifolius*	C	93.5	0.592	pods	brown							x	
Lauraceae	*Actinodaphne angustifolia*	U	36	0.510	berry	black								x
Pentaphylacaceae	*Adinandra integerrima*	U	51.5	0.560	berry	red		x		x				
Meliaceae	*Aglaia edulis*	U	41.6	0.710	capsule	cream			x					
Meliaceae	*Aglaia elaeagnoidea*	LU	28.8	0.630	berry	yellow	x	x						
Meliaceae	*Aglaia lawii*	C	79	0.595	capsule	brown			x					
Meliaceae	*Aglaia spectabilis*	U	26.7	0.575	capsule	orange								x
Rubiaceae	*Aidia densiflora*	U	44.2	0.753	berry	red	x		x	x				
Simaroubaceae	*Ailanthus triphysa*	U	37.1	0.358	samara	brown							x	
Cornaceae	*Alangium chinense*	U	27	0.402	berry	black					X			
Fabaceae-caesalpinioideae	*Albizia attopeuensis*	U	46.4	0.544	pods	dark brown							x	
Euphorbiaceae	*Alchornea rugosa*	LU	18.7	0.412	capsule	brown				x				

Family	Species	Str	Max dbh	WSG	Fruit types	Ripe color	Gi	Ma	Ho	SB	An	TM	Wi	Un
Euphorbiaceae	*Alchornea tiliifolia*	LU	20.7	0.412	capsule	brown				x				
Annonaceae	*Alphonsea boniana*	C	95.7	0.738	monocarp	yellow	x	x						
Apocynaceae	*Alstonia scholaris*	U	41.7	0.382	capsule	brown							x	
Altingiaceae	*Altingia excelsa*	E	126	0.653	synocarp	brown							x	
Rubiaceae	*Anthocephalus chinensis*	C	78.5	0.635	pseudocarp	yellow-brown	x	x						
Moraceae	*Antiaris toxicaria*	C	57.5	0.383	drupe	dark red	x	x	x	x		x		
Phyllanthaceae	*Antidesma montanum*	LU	16.3	0.590	drupe	dark red					x			
Meliaceae	*Aphanamixis polystachya*	U	36.6	0.577	capsule	creamy pink			x					
Ulmaceae	*Aphananthe cuspidata*	E	90.7	0.690	drupe	yellow	x	x	x					
Icacinaceae	*Apodytes dimidiata*	U	50.1	0.610	drupe	black				x				
Euphorbiaceae	*Aporosa octandra*	LU	14.2	0.370	capsule	pink				x				
Euphorbiaceae	*Aporosa octandra* var. *yunnanensis*	LU	13.9	0.370	capsule	brown				x				
Thymelaeaceae	*Aquilaria crassna*	C	56.9	0.347	capsule	light green	x			x				
Fabaceae-mimosoideae	*Archidendron lucidum*	U	28	0.413	pods	red-brown							x	
Myrsinaceae	*Ardisia nervosa*	U	30.8	0.598	drupe	black					x			

Family	Species	Str	Max dbh	WSG	Fruit types	Ripe color	Gi	Ma	Ho	SB	An	TM	Wi	Un
Myrsinaceae	*Ardisia sanguinolenta*	LU	14.3	0.598	drupe	black	x			x				
Arecaceae	*Areca triandra*	LU	5.2	0.569	drupe	red		x	x					
Moraceae	*Artocarpus nitidus*	LU	20.2	0.480	multiple fruit	yellow	x	x						
Phyllanthaceae	*Baccaurea ramiflora*	U	37	0.624	berry	yellow	x	x						
Euphorbiaceae	*Balakata baccata*	E	115	0.509	capsule	black	x	x						
Lauraceae	*Beilschmiedia* aff. *intermedia*	U	58.5	0.573	drupe	black			x					
Lauraceae	*Beilschmiedia glauca*	U	67.3	0.573	drupe	black			x					
Lauraceae	*Beilschmiedia maingayi*	C	62	0.573	drupe	black	x	x	x	x				
Celastraceae	*Bhesa robusta*	C	55.4	0.655	capsule	yellow			x					
Phyllanthaceae	*Bridelia insulana*	U	52	0.542	drupe	dark red	x	x	x	x		x		
Anacardiaceae	*Buchanania arborescens*	U	51.9	0.451	drupe	black	x			x				
Callophyllaceae	*Calophyllum dryobalanoides*	U	43.6	0.650	berry	yellow					x			
Clusiaceae	*Calophyllum polyanthum*	LU	9.4	0.439	berry	yellow								x
Callophyllaceae	*Calophyllum saigonenese*	LU	16.8	0.578	drupe	yellow								x
Theaceae	*Camellia oleifera*	LU	15.7	0.550	capsule	red-brown			x					

Family	Species	Str	Max dbh	WSG	Fruit types	Ripe color	Gi	Ma	Ho	SB	An	TM	Wi	Un
Burseraceae	*Canarium euphyllum*	E	105	0.350	drupe	black			x			x		
Rubiaceae	*Canthium coffeoides*	LU	11.6	0.695	drupe	yellow	x							
Rubiaceae	*Canthium glabrum*	U	26.2	0.465	drupe	black	x		x					
Rhizophoraceae	*Carallia brachiata*	C	86.2	0.666	berry	red	x	x	x	x				
Arecaceae	*Caryota mitis*	LU	11.2	0.569	drupe	purple-black					x			
Salicaceae	*Casearia grewiifolia*	U	45.3	0.491	capsule	yellow				x				
Fagaceae	*Castanopsis acuminatissima*	C	84.9	0.584	capsule	dark brown					x			
Cannabaceae	*Celtis tetrandra*	LU	20.6	0.520	drupe	red	x	x	x					
Euphorbiaceae	*Chaetocarpus castanocarpus*	U	45.1	0.750	capsule	brown				x				
Oleaceae	*Chionanthus ramiflorus*	U	52.2	0.753	drupe	black	x		x					
Meliaceae	*Chisocheton cumingianus*	U	52.7	0.545	capsule	orange-red					x			
Meliaceae	*Chisocheton dysoxylifolius*	C	59.5	0.499	capsule	orange				x				
Anacardiaceae	*Choerospondias axillaris*	C	113	0.487	drupe	yellow	x	x				x		
Meliaceae	*Chukrasia tabularis*	C	73.5	0.548	capsule	brown							x	
Lauraceae	*Cinnamomum ilicioides*	E	185	0.474	drupe	black								x

Family	Species	Str	Max dbh	WSG	Fruit types	Ripe color	Gi	Ma	Ho	SB	An	TM	Wi	Un
Lauraceae	*Cinnamomum iners*	U	17.5	0.499	drupe	black		x		x				
Lauraceae	*Cinnamomum* sp.	U	20.1	0.474	drupe	black					x			
Lauraceae	*Cinnamomum subavenium*	C	63.7	0.500	drupe	black	x	x	x	x		x		
Euphorbiaceae	*Claoxylon indicum*	U	28.5	0.355	capsule	light green				x				
Clusiaceae	*Cratoxylum cochinchinense*	U	27.3	0.670	capsule	brown							x	
Crypteroniaceae	*Crypteronia paniculata*	LU	21.2	0.664	capsule	black					x			
Fabaceae-papilionoideae	*Dalbergia cochinchinensis*	E	129	0.880	pods	dark brown							x	
Daphniphyllaceae	*Daphniphyllum beddomei*	U	50.2	0.511	drupe	black				x				
Annonaceae	*Dasymaschalon acuminatum*	LU	11.4	0.565	monocarp	orange-red			x	x				
Myrtaceae	*Decaspermum parviflorum*	LU	14	0.722	berry	black		x			x			
Urticaceae	*Dendrocnide stimulans*	U	39	0.207	achene	pink				x				
Ebenaceae	*Diospyros glandulosa*	U	49.7	0.697	berry	yellow	x	x				x		
Dipterocarpaceae	*Dipterocarpus gracilis*	E	138	0.603	samaroid	red							x	
Liliaceae	*Dracaena elliptica*	LU	12.4	0.490	berry	yellow	x							
Lythraceae	*Duabanga grandiflora*	C	86	0.379	capsule	black							x	

**Family**	**Species**	**Str**	**Max dbh**	**WSG**	**Fruit types**	**Ripe color**	**Gi**	**Ma**	**Ho**	**SB**	**An**	**TM**	**Wi**	**Un**
Meliaceae	*Dysoxylum cyrtobotryum*	U	43.1	0.591	capsule	orange			x					
Meliaceae	*Dysoxylum arborescens*	C	93.5	0.470	capsule	pink	x		x					
Elaeocarpaceae	*Elaeocarpus floribundus*	LU	11.5	0.580	drupe	purple-black								
Elaeocarpaceae	*Elaeocarpus griffithii*	U	40.5	0.489	drupe	purple-black	x	x						
Elaeocarpaceae	*Elaeocarpus petiolatus*	U	43.3	0.455	drupe	purple-black								x
Elaeocarpaceae	*Elaeocarpus sphaericus*	C	63.2	0.327	drupe	purple-black	x	x				x		
Juglandaceae	*Engelhardia spicata*	C	37.5	0.493	nut	brown							x	
Rosaceae	*Eriobotrya bengalensis*	LU	24.2	0.730	pome	red					x			
Fabaceae-papilionoideae	*Erythrina subumbrans*	C	98.8	0.230	pods	dark brown							x	
Myrtaceae	*Eugenia cerasoides*	E	134	0.724	drupe	dark purple	x			x				
Myrtaceae	*Eugenia grandis*	C	61.8	0.710	drupe	green				x				
Myrtaceae	*Eugenia grata*	U	40	0.724	berry	black					x			
Myrtaceae	*Eugenia lineata*	LU	11.9	0.724	berry	dark purple					x			
Myrtaceae	*Eugenia siamensis*	U	30.2	0.724	berry	yellow	x	x						
Myrtaceae	*Eugenia syzygioides*	C	67	0.750	berry	dark red	x	x		x				

Family	Species	Str	Max dbh	WSG	Fruit types	Ripe color	Gi	Ma	Ho	SB	An	TM	Wi	Un
Celastraceae	*Euonymus glaber*	C	67.2	0.554	capsule	red								x
Pentaphylacaceae	*Eurya acuminata*	U	NA	0.500	berry	black		x		x				
Pentaphylacaceae	*Eurya nitida*	U	18.9	0.530	berry	dark purple		x		x				
Euphorbiaceae	*Excoecaria oppositifolia*	U	39.3	0.485	capsule	brown								x
Moraceae	*Ficus pubilimba*	E	233	0.473	figs	yellow-red	x	x	x	x				
Moraceae	*Ficus annulata*	C	108	0.330	figs	pale yellow	x	x		x				
Moraceae	*Ficus concinna*	C	50.3	0.411	figs	drak pink	x	x		x				
Moraceae	*Ficus fistulosa*	LU	20.5	0.380	figs	yellow					x			
Moraceae	*Ficus glaberrima*	C	45.5	0.411	figs	cream	x	x		x				
Moraceae	*Ficus hispida*	LU	19.5	0.382	figs	yellow-brown					x			
Moraceae	*Ficus kurzii*	C	161	0.411	figs	dark violet	x	x	x	x				
Moraceae	*Ficus lamponga*	C	48.6	0.411	figs	orange					x			
Moraceae	*Ficus microcarpa*	U	38.2	0.411	figs	pink	x	x	x	x				
Moraceae	*Ficus nervosa*	C	100	0.280	figs	yellow	x		x	x				
Moraceae	*Ficus pubilimba*	C	—	0.411	figs	yellow-red	x			x				

Family	Species	Str	Max dbh	WSG	Fruit types	Ripe color	Gi	Ma	Ho	SB	An	TM	Wi	Un
Moraceae	*Ficus stricta*	C	2.6	0.411	figs	orange	x	x	x	x				
Moraceae	*Ficus subcordata*	C	—	0.411	figs	orange	x			x				
Moraceae	*Ficus tinctoria*	C	—	0.411	figs	orange	x			x				
Moraceae	*Ficus triloba*	LU	26.9	0.411	figs	orange	x	x	x					
Moraceae	*Ficus variegata*	C	55.4	0.327	figs	dark brown	x	x	x	x				
Moraceae	*Ficus vasculosa*	C	43.5	0.300	figs	yellow	x	x	x	x				
Oleaceae	*Fraxinus floribunda*	C	71.6	0.545	samara	brown							x	
Clusiaceae	*Garcinia benthamii*	U	44.5	0.736	berry	yellow	x	x				x		
Clusiaceae	*Garcinia eugeniaefolia*	LU	4.5	0.736	berry	yellow								x
Cannabaceae	*Gironniera nervosa*	C	72.1	0.450	drupe	orange	x	x		x				
Phyllanthaceae	*Glochidion assamicum*	LU	23.8	0.563	capsule	red				x	x			
Phyllanthaceae	*Glochidion eriocarpum*	LU	—	0.563	capsule	dull red								
Phyllanthaceae	*Glochidion rubrum*	U	26.4	0.640	capsule	red		x		x				
Stemonuraceae	*Gomphandra tetrandra*	U	29.5	0.456	drupe	black	x							
Cardiopteridaceae	*Gonocaryum lobbianum*	U	28.2	0.662	drupe	black								x

Family	Species	Str	Max dbh	WSG	Fruit types	Ripe color	Gi	Ma	Ho	SB	An	TM	Wi	Un
Proteaceae	*Helicia formosana*	U	42.9	0.608	berry	brown					x			
Malvaceae	*Hibiscus macrophyllus*	U	31.9	0.375	capsule	brown				x				
Salicaceae	*Homalium cochinchinense*	U	22.8	0.711	capsule	brown				x				
Dipterocarpaceae	*Hopea ferrea*	C	80	0.890	wing nut	pink-orange							x	
Dipterocarpaceae	*Hopea odorata*	C	82	0.635	wing nut	brown							x	
Myristicaceae	*Horsfieldia amygdalina*	U	55	0.460	capsule	yellow			x					
Achariaceae	*Hydnocarpus castanea*	U	48.2	0.700	capsule	yellow-brown			x	x				
Achariaceae	*Hydnocarpus ilicifolius*	LU	21.3	0.810	capsule	dark brown					x			
Aquifoliaceae	*Ilex* aff. *chapaensis*	U	43.5	0.564	drupe	red				x				
Aquifoliaceae	*Ilex chevalieri*	C	64.8	0.564	drupe	red	x			x				
Myristicaceae	*Knema elegans*	U	34.9	0.533	capsule	brown	x	x	x					
Myristicaceae	*Knema globularis*	U	—	0.533	capsule	light brown								
Lythraceae	*Lagerstroemia calyculata*	U	41.8	0.720	capsule	dark brown							x	
Lauraceae	*Lindera communis*	U	42.8	0.515	drupe	red				x				
Fagaceae	*Lithocarpus ceriferus*	U	29.7	0.668	nut	brown					x			

Family	Species	Str	Max dbh	WSG	Fruit types	Ripe color	Gi	Ma	Ho	SB	An	TM	Wi	Un
Fagaceae	*Lithocarpus eucalyptifolius*	U	47.1	0.668	nut	brown					x			
Lauraceae	*Litsea beusekomii*	C	75.1	0.425	drupe	black				x				
Lauraceae	*Litsea martabanica*	LU	17.4	0.425	drupe	black					x			
Lauraceae	*Litsea monopetala*	U	51.7	0.423	berry	black		x		x				
Lauraceae	*Litsea umbellata*	LU	18.2	0.340	drupe	black					x			
Lauraceae	*Litsea verticillata*	LU	26.4	0.425	drupe	black					x			
Celastraceae	*Lophopetalum wightianum*	U	42.7	0.370	capsule	brown							x	
Euphorbiaceae	*Macaranga denticulata*	U	37.6	0.434	capsule	brown				x				
Euphorbiaceae	*Macaranga siamensis*	C	60.9	0.381	capsule	brown				x				
Araliaceae	*Macropanax dispermus*	LU	—	0.509	berry	black								x
Lauraceae	*Machilus* aff. *salicina*	C	71.1	0.559	berry	black					x			
Lauraceae	*Machilus gamblei*	C	62.7	0.559	berry	black					x			
Magnoliaceae	*Magnolia baillonii*	E	111	0.500	aggregat e	green-brown				x				
Euphorbiaceae	*Mallotus paniculatus*	U	30.1	0.345	capsule	red-brown				x				
Euphorbiaceae	*Mallotus philippensis*	U	24.8	0.603	capsule	red-brown				x				

Family	Species	Str	Max dbh	WSG	Fruit types	Ripe color	Gi	Ma	Ho	SB	An	TM	Wi	Un
Euphorbiaceae	*Mallotus resinosus*	LU	10	0.503	capsule	green								x
Anacardiaceae	*Mangifera cochinchinensis*	C	62.2	0.513	drupe	yellow		x				x		
Anacardiaceae	*Mangifera duperreana*	C	59.8	0.517	drupe	yellow		x				x		
Bignoniaceae	*Markhamia stipulata*	U	43	0.676	capsule	brown							x	
Nyssaceae	*Mastixia pentandra*	C	66.5	0.495	drupe	purple-black			x					
Sabiaceae	*Meliosma pinnata*	C	52.1	0.320	capsule	dark brown								x
Sabiaceae	*Meliosma simplicifolia*	U	27.8	0.455	drupe	black					x			
Melastomataceae	*Memecylon edule*	LU	12.1	0.675	berry	black				x				
Melastomataceae	*Memecylon lilacinum*	U	22	0.820	berry	black	x			x				
Melastomataceae	*Memecylon ovatum*	LU	6.9	0.768	berry	black				x				
Rubiaceae	*Metadina trichotoma*	LU	29.9	0.720	capsule	dark purple								x
Annonaceae	*Miliusa lineata*	U	58	0.650	aggregate	red	x	x						
Sapindaceae	*Mischocarpus pentapetalus*	U	25.5	0.731	capsule	yellow-green				x				
Moraceae	*Morus macroura*	U	39.6	0.550	achene	purple-black					x			
Rubiaceae	*Nauclea orientalis*	U	48.1	0.483	pseudocarp	yellow-brown	x	x						

Family	Species	Str	Max dbh	WSG	Fruit types	Ripe color	Gi	Ma	Ho	SB	An	TM	Wi	Un
Lauraceae	*Neocinnamomum mekongense*	LU	16.8	0.559	drupe	red					x			
Lauraceae	*Neolitsea zeylanica*	LU	23.3	0.533	drupe	red					x			
Sapindaceae	*Nephelium melliferum*	C	76	0.762	drupe	red	x	x						
Icacinaceae	*Nothapodytes montana*	U	22	0.577	drupe	red		x			x			
Oleaceae	*Olea brachiata*	LU	21.2	0.630	drupe	purple-black					x			
Fabaceae-papilionoideae	*Ormosia sumatrana*	C	66.8	0.593	pods	brown				x				
Bignoniaceae	*Oroxylum indicum*	U	33.8	0.411	flat capsule	dark greyish brown							x	
Sapotaceae	*Palaquium garrettii*	U	42.5	0.580	drupe	orange	x	x						
Lauraceae	*Phoebe cathia*	C	94.2	0.544	drupe	black	x	x	x	x		x		
Lauraceae	*Phoebe lanceolata*	U	55.5	0.689	drupe	black			x	x				
Arecaceae	*Pinanga sylvestris*	LU	6.4	0.569	drupe	red					x			
Icacinaceae	*Platea latifolia*	C	76.3	0.340	drupe	dark purple	x	x	x	x				
Annonaceae	*Platymitra macrocarpa*	E	98.7	0.610	monocarp	pale brown	x	x				x		
Podocarpaceae	*Podocarpus imbricatus*	E	128	0.478	drupe	orange					x			
Podocarpaceae	*Podocarpus neriifolius*	C	81.9	0.477	berry	dark purple	x		x	x				

**Family**	**Species**	**Str**	**Max dbh**	**WSG**	**Fruit types**	**Ripe color**	**Gi**	**Ma**	**Ho**	**SB**	**An**	**TM**	**Wi**	**Un**
Annonaceae	*Polyalthia* aff. *evecta*	LU	13	0.567	aggregate	dark purple	x			x				
Annonaceae	*Polyalthia simiarum*	U	47.9	0.594	aggregate	dark purple	x	x	x					
Polyosmaceae	*Polyosma* cf. *integrifolia*	U	34	0.530	drupe	purple	x	x		x				
Sapotaceae	*Pouteria stellibacca*	E	105	0.714	berry	yellow-red	x	x	x					
Rosaceae	*Prunus arborea*	U	53	0.456	drupe	black	x			x				
Rosaceae	*Prunus javanica*	E	114	0.670	drupe	yellow	x	x	x					
Sterculiaceae	*Pterocymbium tinctorium*	C	60.5	0.250	papery follicle	light brown							x	
Sterculiaceae	*Pterospermum cinnamomeum*	C	86.5	0.521	capsule	brown							x	
Fagaceae	*Quercus quangtriensis*	C	55.7	0.701	nut	brown					x			
Bignoniaceae	*Radermachera ignea*	LU	21	0.483	pods	brown							x	
Myrsinaceae	*Rapanea yunnanensis*	U	32.8	0.650	berry	black					x			
Anacardiaceae	*Rhus rhetsoides*	U	49.5	0.585	drupe	greyish purple				x				
Meliaceae	*Sandoricum koetjape*	C	75.5	0.473	berry	orange	x	x				x		
Sapotaceae	*Sarcosperma arboreum*	C	82.4	0.413	drupe	black violet			x					
Actinidiaceae	*Saurauia roxburghii*	LU	24	0.579	berry	white	x	x		x				

Family	Species	Str	Max dbh	WSG	Fruit types	Ripe color	Gi	Ma	Ho	SB	An	TM	Wi	Un
Araliaceae	*Schefflera heptaphylla*	U	57	0.427	berry	black	x	x		x				
Theaceae	*Schima wallichii*	E	103	0.558	capsule	brown							x	
Santalaceae	*Scleropyrum pentanarum*	U	32.2	0.725	drupe	yellow								x
Dipterocarpaceae	*Shorea henryana*	C	78.5	0.660	wing nut	brown							x	
Elaeocarpaceae	*Sloanea sigun*	C	82.5	0.490	capsule	light green			x	x				
Sterculiaceae	*Sterculia balanghas*	U	36.7	0.426	capsule	dark yellow			x	x				
Sterculiaceae	*Sterculia guttata*	U	42.1	0.426	capsule	orange			x	x				
Symplocaceae	*Symplocos cochinchinensis*	C	56	0.515	drupe	black	x	x	x	x				
Symplocaceae	*Symplocos sumuntia*	U	22	0.536	drupe	black		x		x				
Rubiaceae	*Tarennoidea wallichii*	U	42.1	0.635	berry	black								x
Pentaphylacaceae	*Ternstroemia wallichiana*	U	32.8	0.618	capsule	yellow			x					
Rutaceae	*Tetradium glabrifolium*	C	59.3	0.232	capsule	brown				x				
Tetramelaceae	*Tetrameles nudiflora*	E	66.8	0.307	capsule	brown							x	
Meliaceae	*Toona ciliata*	E	91.4	0.376	capsule	black							x	
Cannabaceae	*Trema orientalis*	U	36.9	0.345	drupe	dark violet				x				

Family	Species	Str	Max dbh	WSG	Fruit types	Ripe color	Gi	Ma	Ho	SB	An	TM	Wi	Un
Euphorbiaceae	*Triadica cochinchinensis*	U	45.3	0.509	capsule	dark red								x
Tapisciaceae	*Turpinia cochinchinensis*	U	25.5	0.394	drupe	black							x	
Adoxaceae	*Viburnum cylindricum*	LU	20.9	0.631	drupe	red				x				
Lamiaceae	*Vitex quinata*	U	45.5	0.451	drupe	yellow					x			
Meliaceae	*Walsura pinnata*	LU	1.1	0.868	berry	yellow								x
Meliaceae	*Walsura robusta*	U	47.5	0.868	berry	yellow	x	x						
Polygalaceae	*Xanthophyllum virens*	U	47.7	0.704	drupe	yellow					x			

Table S2 Results of statistical tests in maximum tree size (Size) and mean wood specific gravity (WSG) between tree species dispersed by LBF and the other agent in the two types as categorized for defaunation scenarios, all LBF extirpated and only primates extirpated.

	Size		WSG	
Statistics	All Large	Only primates	All Large	Only Primates
t	1.35	-0.943	2.084	2.01
P (t-test)	0.178	0.347	0.038	0.046
F	0.771	0.752	0.75	0.89
P (F-test)	0.274	0.464	0.226	0.799

Table S3 Results of the simulation **without** the assumption of size structure constant for the defaunation of all large-bodied frugivores, the defaunation of all primate and control scenario: median with lower and upper boundary of 95% confidence interval estimated by the bootstrapping method across the gradient of defaunation (%intensity of defaunation).

%intensity of defaunation	% Above-ground carbon loss								
	All LBF extirpated			Only primates extirpated			Control		
	Median	Lower	Upper	Median	Lower	Upper	Median	Lower	Upper
20.00	-0.38	-0.75	0.09	-0.13	-0.30	0.17	0.07	-0.39	0.58
40.00	-1.28	-1.98	-0.50	-0.78	-1.11	-0.35	-0.03	-0.60	0.63
60.00	-1.81	-2.65	-0.99	-1.74	-2.21	-1.21	0.19	-0.69	0.84
80.00	-1.78	-2.63	-0.89	-1.78	-2.22	-1.24	0.11	-0.55	0.87
100.00	-3.02	-3.88	-2.03	-2.31	-2.83	-1.66	0.24	-0.64	1.06

Table S4 The same as TableS4 (**without** the assumption of size structure constant), but here demonstrates actual values of carbon loss (or increase) when relatively compare with the present total above-ground biomass (4599.2 ton Carbon)

%intensity of defaunation	Above-ground carbon loss (ton) in the 30-ha plot								
	All LBF extirpated			Only primates extirpated			Control		
	Median	Lower	Upper	Median	Lower	Upper	Median	Lower	Upper
20	-17.3	-34.2	4.1	-5.9	-13.7	7.8	3.2	-17.8	26.4
40	-58.4	-90.3	-22.8	-35.6	-50.6	-16.0	-1.4	-27.4	28.7
60	-82.5	-120.8	-45.1	-79.3	-100.8	-55.2	8.7	-31.5	38.3
80	-81.2	-119.9	-40.6	-81.2	-101.2	-56.5	5.0	-25.1	39.7
100	-137.7	-176.9	-92.6	-105.3	-129.0	-75.7	10.9	-29.2	48.3

Table S5 Results of the simulation with the assumption of size structure constant for the defaunation of all large-bodied frugivores, the defaunation of all primate and control scenario: median with lower and upper boundary of 95% confidence interval estimated by the bootstrapping method across the gradient of defaunation (%intensity of defaunation).

%intensity of defaunation	% Above-ground carbon loss								
	All LBF extirpated			Only primates extirpated			Control		
	Median	Lower	Upper	Median	Lower	Upper	Median	Lower	Upper
20	-0.40	-0.58	-0.22	-0.49	-0.57	-0.39	0.14	-0.01	0.33
40	-0.83	-1.09	-0.61	-0.85	-0.96	-0.73	0.02	-0.25	0.26
60	-1.14	-1.47	-0.84	-1.38	-1.54	-1.22	0.08	-0.18	0.37
80	-1.50	-1.80	-1.20	-1.86	-2.04	-1.68	0.30	-0.03	0.66
100	-2.35	-2.75	-2.03	-2.39	-2.62	-2.18	0.37	0.04	0.71

Table S6 The same as TableS5 (the assumption of size structure constant), but here demonstrates actual values of carbon loss (or increase) when relatively compare with the present total above-ground biomass (4599.2 ton Carbon)

**%intensity of defaunation**	**Above-ground carbon loss (ton) in the 30-ha plot**								
	**All LBF extirpated**			**Only primates extirpated**			**Control**		
	**Median**	**Lower**	**Upper**	**Median**	**Lower**	**Upper**	**Median**	**Lower**	**Upper**
20	-18.2	-26.4	-10.0	-22.3	-26.0	-17.8	6.4	-0.5	15.0
40	-37.8	-49.7	-27.8	-38.8	-43.8	-33.3	0.9	-11.4	11.9
60	-52.0	-67.0	-38.3	-62.9	-70.2	-55.6	3.6	-8.2	16.9
80	-68.4	-82.1	-54.7	-84.8	-93.0	-76.6	13.7	-1.4	30.1
100	-107.1	-125.4	-92.6	-109.0	-119.5	-99.4	16.9	1.8	32.4
